# Severe asthma in children: An official statement from Saudi Pediatric Pulmonology Association

**DOI:** 10.15537/smj.2022.4.43.20210756

**Published:** 2022-04

**Authors:** Adel S. Alharbi, Abdullah A. Yousef, Saleh A. Alharbi, Talal M. Almaghamsi, Mansour M. Al Qwaiee, Faisal M. Al-Somali, Turki S. Alahmadi, Sami A. Alhaider, Wadha H. Alotaibi, Mona A. Albalawi, Faisal N. Alotaibi, Ahmed S. Alenizi, Muslim M. Alsaadi, Yazan S. Said

**Affiliations:** *From the Department of Pediatrics (A. Alharbi, Alotaibi), Pediatric Pulmonology Division and Pediatric Sleep Center, from the Department of Pediatrics (Al-Somali), Pediatric Pulmonary Division, Prince Sultan Military City, from the Departments of Pediatric Pulmonology & Sleep Medicine (Albalawi), King Fahad Medical City, from the Pediatric Pulmonology And Sleep Medicine Department (Alenizi), Children’s Hospital, King Saud Medical City, from the Department of Pediatrics (Alenizi), College of Medicine and King Khalid University Hospital, King Saud University, From the Pediatric Department (Said), Security Forces Hospital, Riyadh; from the Department of Pediatrics (Yousef), Imam Abdulrahman Bin Faisal University, College of Medicine; from the Department of Pediatrics (Almaghamsi, Alhaider), King Fahad Specialist Hospital, Dammam; King Fahd Hospital of the University (Yousef), from the Department of Pediatrics (Alahmadi), Dr. Sulaiman Al Habib Hospital, Al-Khobar; Department of Pediatrics (S. Alharbi), Faculty of Medicine, Umm Alqura University, Mecca; from the Department of Pediatrics (S. Alharbi), Dr. Soliman Fakeeh Hospital; from the Pediatric Department (Al Qwaiee), King Faisal specialist hospital & Research Center, from the Department of Pediatrics (Alotaibi), Faculty of Medicine, King Abdulaziz University, Jeddah, Kingdom of Saudi Arabia.*

**Keywords:** severe asthma, pediatric asthma, Saudi Arabia, task force

## Abstract

In Saudi Arabia, the prevalence of pediatric asthma ranges between 8% and 25%. However, there are no sufficient data regarding severe asthma in childhood in Saudi Arabia. Therefore, a task force has been formed by the Saudi Pediatric Pulmonology Association which is a subsidiary group of the Saudi Thoracic Society and consists of Saudi experts with well-respected academic and clinical backgrounds in the fields of pediatric asthma as well as other respiratory diseases to write a consensus on definitions, phenotypes, and pathophysiology, evaluation, and management. To achieve this, the subject was divided into various sections, each of which was assigned to at least 2 experts. Without a central literature review, the authors searched the literature using their own strategies. To reach an agreement, the entire panel reviewed and voted on proposed findings and recommendations.

## 1. Introduction

Asthma is the most common chronic lower respiratory disease that commonly begins in childhood and has a wide range of symptoms and phenotypes that can progress or subside over time.^1^ Only 2 to 5% of children have severe asthma; however, its burden on the economy and resource usage is significant.^1-3^ Even though most asthmatic children can be effectively treated with currently available drugs, many asthmatic children remain difficult-to-treat (DTA).^4^ Much remains unknown on the optimal techniques for treating these patients. Unlike adults, children with severe asthma have higher total serum immunoglobulin E (IgE), increased blood eosinophils, and multiple aeroallergen sensitization.^5^ The main comorbidities detected in pediatrics were bronchial hyperresponsiveness (BHR) and decreased lung function.^6-8^

The Saudi Pediatric Pulmonology Association (SPPA)-Pediatric Severe Asthma Task Force includes clinicians with expertise in severe asthma, representing most Saudi health authorities. The task force decided to write a consensus on definitions, phenotypes, pathophysiology, evaluation, and management of severe asthma with a specific recommendation for practice. The methods employed in this document to develop clinical recommendations follow local and worldwide guidelines. The task force provides the basis for rational decisions in managing severe asthma according to international standards.

## 2. Methods

The task force consisted of 14 invited pediatric asthma experts. The subject was initially subdivided into many topics. At least, 2 specialists were selected for each topic. Topic writers carried out their own literature searches and created their own databases based on the results of those searches. There was no attempt to assess the evidence or the recommendations. Experts were provided opportunities to have their ideas heard and considered by their peers through the use of the nominal group technique (NGT), an organized face-to-face group interaction.^8^ As of July 2021, the literature search was completed and the findings were presented. There were 2 virtual sessions held in April and July 2021 in which the experts provided draft reports and received feedback from the rest of the panel. The whole panel examined and discussed the recommendations and supporting evidence during these meetings. A consensus was necessary for the recommendations to be approved, and that was defined as a majority approval. The recommendations were updated several times until everyone agreed with them. Although the guidelines, medications, and technologies on the market varied, the panel made an effort to produce a consensus statement that would be applicable worldwide.

## 3. Definition

The severity assessment of asthmatic children in the clinical setting is essential as it guides the management plan and determines the need for referral to a specialist.^9^ The asthma severity assessment is dictated by the treatment step needed to control the patient’s symptoms.^1^ Even though severe asthma has multiple definitions, the differences between them are subtle.^10^ The recent guidelines by European Respiratory Society/American Thoracic Society and Global Initiative for Asthma define it as “asthma that requires step 4 or 5 therapy (high-dose inhaled corticosteroids [ICS]) plus a second controller)- to be controlled or uncontrolled”.^11^ A study discovered that 4.5% of children diagnosed with asthma had “severe asthma,” with an estimated prevalence of 0.5%.^12^ Difficult-to-treat asthma is defined as uncontrolled asthma related to a poor inhaler technique, suboptimal adherence to therapy, untreated modifiable factors, or an incorrect diagnosis of asthma.^13^ Labels such as “refractory asthma” and “treatment-resistant asthma” are no longer appropriate with the emergence of biological therapies.^14^

## 4. Burden and epidemiology of severe asthma

Asthma remains a prevalent global health and socio-economic problem, despite several decades of progress in asthma management. Severe asthma risk factors have been identified on the basis of several epidemiological studies. Though severe asthma in children occasionally presents during school age; however, it tends to start earlier (in the first 3 years of life) in those with severe asthma compared to those with mild-to-moderate asthma who tend to have their symptoms onset relatively later (5 years of age or later).^15,16^ Babies born with lower lung function, assessed by maximal expiratory flows at functional residual capacity (Vmax [FRC]), shortly after birth have a higher risk of severe childhood asthma.^17,18^ Atopic dermatitis, bronchial hyperresponsiveness, airway obstruction, high fractional exhaled nitric oxide (FeNO), and African American race are all risk factors for severe childhood asthma.^16,19-23^

There are no sufficient data on severe asthma in childhood in Saudi Arabia. Recent investigations in Saudi Arabia demonstrated that between 1986 and 2017, the prevalence of childhood asthma varied across the country, from 9% in the Southern region to 33.7% in the Eastern region.^23^ According to the Saudi Initiative for Asthma, children in Saudi Arabia have an asthma prevalence rate of 8 to 25%.^24^ This discrepancy could be explained by the different surveying methods that were used during the assessment of prevalence or the different age groups that were assessed. Approximately 30% of Saudi Arabian citizens are under the age of 15, and 68% fall somewhere between 15 and 64.^25^ As a result, childhood asthma is likely to remain a serious public health concern in Saudi Arabia.

Chronic symptoms, acute exacerbations, and drug side effects are common in patients with severe asthma. Patients with severe asthma may experience disruptions in their ordinary activities, sleep, physical activity, social life, and mental health.^26^ Severe asthma has a significant financial impact on society.^27^ The total cost of asthma in the United States in 2013, based on the pooled sample, was $81.9 billion, including expenses associated with absenteeism and mortality.^28^ The most significant cost drivers of direct costs were discovered to be hospitalization and drugs.^29^ Controlling asthma has the ability to enhance not just one’s health but also save money on hospital costs and increase productivity. More research is needed to determine the prevalence of severe childhood asthma and its burden on the healthcare system in Saudi Arabia.

## 5. Pathogenesis of severe asthma

Asthma has been known to be an eosinophilic airway inflammatory disease linked to BHR.^30-32^ Indeed, the quantity of eosinophils in the lungs is correlated with the severity of the disease and has been used to classify clinical phenotypes and guide treatment in severe asthma.^33^ The immunopathogenesis of severe asthma is different from mild to moderate asthma as there are significant differences in the immune response and the extent and type of subsequent inflammatory cytokine production.^33^ Another subset of severe asthma is glucocorticoid-resistant asthma, which occurs due to multiple pathophysiological mechanisms.^34^

The inflammatory cascade in severe asthma is mainly caused by T-helper 2 cells (Th-2) activation and the release of Th-2 related cytokines, predominantly Interleukin-4 (IL-4), IL-5, and IL-13.^32,34^ The extent of expression of these cytokines correlates with asthma severity. Moreover, severe asthma is associated with inflammatory responses by other T-helper cells, which are Th-17 and Th-1. The Th-17 response is initiated by IL-6 and maintained by IL-23, which releases IL-17, enhancing the neutrophilic production.^33^ Interferon-gamma (IFN-g) is another cytokine that has been implicated in severe asthma, and it is released through the activation of Th-1 cells. Finally, innate immunity, precisely, innate lymphoid cell type 2, has a major role in severe asthma pathogenesis. Iymphoid cell type 2 mediators include thymic stromal lymphopoietin (TSLP), IL-25, and IL-33. IL-33, in particular, is linked to severe asthma and changes in the airways (Figure 1).^34^

**Figure 1:**
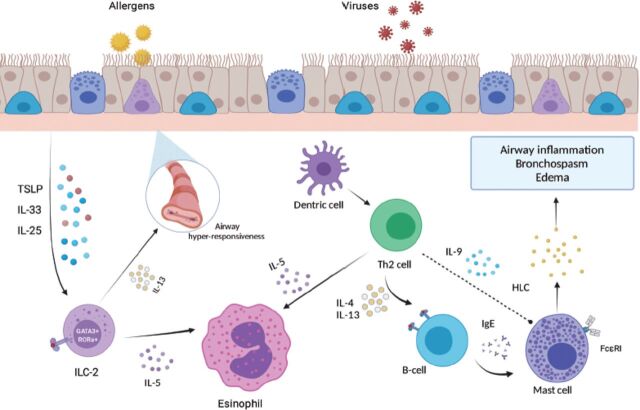
- Pathogenesis of severe asthma. IL: interleukin, TSLP: thymic stromal lymphopoietin, ILC-2: innate lymphoid cell type 2; RORA: related orphan receptor A, Th2: T-helper cell-2, IgE: immunoglobulin E, HLC: histamine-leukotrienes-cytokines.

**Figure 1:**
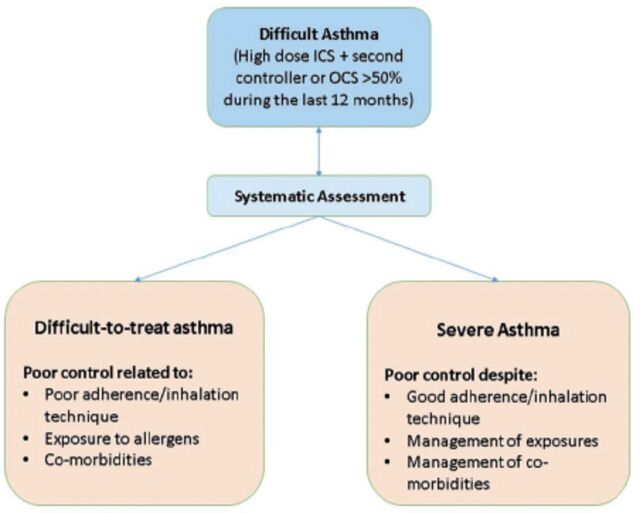
- Definition and classification of difficult asthma ICS: inhaled corticosteroids; OCS: oral corticosteroids

Glucocorticoids (GC) act by binding to the glucocorticoid receptors (GR) in the cytoplasm, forming a complex that binds to the DNA, causing an anti-inflammatory effect. There are 2 subtypes of glucocorticoid receptors, GR-α and GR-α. Normally GC binds to GR-a to elicit the anti-inflammatory reaction. Unlike GR-α, GR-α does not bind to GC and acts as a weak dominant inhibitor of GR-α.^32,34^ Reduced GR-binding affinity, GR-overexpression, decreased histone deacetylase (HDAC) activity, and genetic predisposition all contribute to GC resistance asthma.^35-37^ The reduction in GR binding affinity has been linked to the expression of IL-4 and IL-2.^38^

Meanwhile, the evidence is inconclusive regarding the relationship between GR-α overexpression and severe asthma.^39^ Alternatively, the reduction of HDAC activity is linked to phosphorylation by a phosphoinositide 3-kinase that is activated by oxidative stress.^40,41^ GC-resistant asthma might have a genetic predisposition, and there is speculation that it’s linked to certain genetic variants, in particular, glucocorticoid-induced transcript 1 gene (GLCCI1).^42,43^

### Type 2-low asthma

In the pediatric population, T2-low asthma is less common than T2-high asthma and has not been fully understood yet.^41^ In moderate to severe asthma patients, the activation of Th-1 and Th-17 cells by T2-low asthma is detected.^42^ These patients are usually older, less prone to allergies, and less responsive to corticosteroids.^43^ There has been little progress in the research of therapeutic medications for T2-low asthma. The promising efficacy of azithromycin and bronchial thermoplasty has been reported.^44^

### Type 2-high asthma

Both allergic and non-allergic eosinophilic asthma are classified as T2-high asthma. In allergic asthma, IgE-dependent mechanisms are crucial, while non-allergic asthma may be dominated by T2 cytokine inflammation.^45^ In addition, IL-33, IL-25, and thymine stromal lymphopoietin are activated by the interaction between the airway epithelium and the pollutants, inhaled allergens, and microorganisms in T2-high asthma, leading to further activation of IL-4 and IL-5, enhancing the upregulation of vascular endothelium attachment receptors and participate in the maturity and survival of eosinophils, respectively.^46^ The stimulation of the prostaglandin dopamine receptor causes eosinophils to be attracted to the lung mucous membrane.^47^ Inflammation of the bronchial epithelium causes bronchial obstruction and leukotriene generation.^48^ Immunoglobulin E is produced in B cells by IL-4, and IgE unites with mast cells to induce cell death, which guarantees cytokines and eicosanoids, promoting airway inflammation. Airway smooth muscle hypersensitivity and mucus hypersecretion are also linked to IL-13.^49^ The response to biologics can be predicted by the count of sputum and blood eosinophil, serum periostin, and IgE.

## 6. Evaluation of severe asthma in children

The evaluation of a patient with severe asthma can be challenging. Severe asthma is a heterogeneous and dynamic disease, and a careful approach with temporal observation and follow-up is paramount. With that being said, the main objective of the evaluation is to tease out other causes of problematic asthma,as seen in Figure 2, so we can define outpatients with “true” severe asthma, as most patients who present with what is labeled as “severe” asthma end up not having it.^5,50-53^

The evaluation is usually carried out in specialized centers with a dedicated multidisciplinary team. Each member will have a clear role with usually pre-determined forms and checklists. This guarantees a uniform evaluation and decreases any interpersonal variations. Some centers will also add a home visit to the evaluation.^6,7,54^ The evaluation approach for severe asthma can be simplified into 3 steps:

### Confirm diagnosis of asthma

*Full clinical evaluation:* This is the best and most affordable technique to diagnose asthma. As many asthma mimickers are classified as “severe asthma,” a thorough history is required. The clinical history should be documented. Chronic obstructive pulmonary disease is a common cause of difficulty breathing (DOB).^9^ Similarly, not all wheezes are expiratory noises or indicate airway obstruction. Many people use the phrase “wheeze” to describe any noisy breathing or even DOB. Furthermore, it is imperative to say a direct translation may affect the accuracy of history taking.^11^ For example, many patients have variable bedtimes during holidays. So a “night” cough might arise throughout the day. Try to identify symptoms or events that offer alternate diagnoses. A child “wheezing” since birth, having a year-round wet cough, not responding to bronchodilator, and coughing exclusively during wakefulness is most likely not asthmatics.^53^ A detailed physical examination should follow this, with a focus on the symptoms and signs of other illnesses, like aches and pain. A child with failure to thrive, stridor, and crackles (of course, among others) is most likely not asthmatic. As a result of the detailed history and physical examinations, a personalized action plan will be put in place. This plan will tell you which tests, investigations, and interventions need to be carriedout.^54^

*Pulmonary function tests*: These are used to assess the patient’s degree of airflow limitations, response to bronchodilators, lung volumes, and air trapping, among others. Spirometry should always be carried out to determine severe pediatric asthma with elevated bronchodilator response, which may be linked with impaired lung function.^54,55^ A prolonged bronchodilator reversibility (BDR) may be linked to poor medication compliance or incorrect inhaler technique and may be indicative of a favorable response to ICSs.^54^ Bronchoprovocation tests (such as the methacholine challenge test) can be used if an asthma diagnosis is in question.

*Other investigations:* The European respiratory society suggested that children between the ages of 5 and 16 who are suspected of having asthma should be tested for FeNO.^56^ Table 1 shows a wide range of tests for severe asthma diagnosis.

**Table 1:** - Common tests when assessing severe asthma.

**Diagnostic study**	**Test**
Radiology	• Chest x-ray
	• Chest CT
	• Gastrointestinal assessment (PH/impedance probe, barium studies, or scoping)
	• Bronchoscopy (flexible versus rigid) with Bronchoalveolar lavage
Blood and serological test	• CBC with differential looking for eosinophilia
	• Total IgE
	• Skin prick testing or specific IgE measurement
	• Immunoglobulin level
Others	• Sweat chloride test
	• Spirometry (including post bronchodilatation, lung volumes)
	• Sleep studies
	• Genetic testing

IgE: immunoglobulin E, CBC: complete blood count, CT: computed tomography

### Check barriers for asthma control

*Adherence:* Poor adherence to severe asthma medications is common; it may be difficult to identify non-adherent patients. Therefore, it is crucial to question the patient every time he attends the clinic regarding his compliance and double-check that with his prescription refills, medication counters, and caregiver feedback.^57^

*Techniques:* A very important factor causing the poor response to medication is the inappropriate technique used in administering the medications. Children should never receive metered dose inhaler inhaled directly through the mouth. Proper use of valved holding chambers (spacers) is necessary, and it should be reviewed every visit.^58^

*Environment:* Both home and school environments should be checked for possible indoor allergens triggering asthma, such as house dust mites, pets, mold, smoking, etc. Outdoor allergens such as air pollutants, sandstorms, and plants contribute to the poor control of severe asthma.^59^

### Exclude comorbidities

Comorbidities are often linked to asthma severity and may contribute to poor control. In the Severe Asthma Research Program III cohort of children, body mass index, gastroesophageal reflux disease (GERD), and sinusitis were significantly linked with exacerbation frequency, hence identifying and controlling GERD I is critical.^60^ Allergic rhinitis, adenoid hypertrophy, and obesity should be also controlled.^61^ Table 2 shows a list of common comorbidities that need to be looked for.

**Table 2:** - Comorbidities associated with severe asthma.

• Allergic rhinitis
• Sleep-disordered breathing
• EILO/VCD
• GERD
• Obesity
• Eosinophilic esophagitis
• ABPA

VCD: vocal cord dysfunction, EILO: exercise-induced laryngeal obstruction, GERD: gastroesophageal reflux disease, ABPA: allergic bronchopulmonary aspergillosis

## 7. Biological treatment of severe asthma in children

There are now 5 biological drugs, including Omalizumab, which binds to the high-affinity IgE receptor, Mepolizumab and Reslizumab that bind IL5, Benralizumab which binds to the IL5 receptor subunit, and Dupilumab which attaches to the IL4 receptor subunit and so blocks both IL4 and IL13,^62^ Table 3. Tezepilumab, a TSLP-binding antibody, is now in phase 2B studies. Only 2 (Mepolizumab and Omalizumab) have been approved for use in children with asthma, while dupilumab has been approved for use in children with atopic dermatitis.^63,64^

**Table 3:** - Summary of biologics currently approved for severe asthma

**Drug**	**Dose and route**	**Indication**	**Mechanism of action**
Dupilumab (Dupixent)	400-600 mg SC loading dose followed by 200 or 300 mg SC every 2 wk	≥12 yr old; AEC ≥150 cells/µL or FeNO ≥25 ppb with OCS-dependent	Anti–IL-4R; binds to IL-4 receptor α; blocks signaling of IL-4 and IL-13
Benralizumab (Fasenra)	30 mg SC every 4 wk for three doses; followed by every 8 wk subsequently	≥18 yr old; severe eosinophilic asthma	Anti–IL-5; binds to IL-5 receptor α; causes apoptosis of eosinophils and basophils
Reslizumab (Cinqair)	Weight-based dosing of 3 mg/kg IV every 4 wk	≥18 yr old; AEC ≥400 cells/µL	Anti–IL-5; binds to IL-5 ligand; prevents IL-5 from binding to its receptor
Mepolizumab (Nucala)	100 mg SC every 4 week	≥6 yr old; AEC ≥150 cells/µL or ≥300 cells/µL at least once a year	Anti–IL-5; binds to IL-5 ligand; prevents IL-5 from binding to its receptor
Omalizumab (Xolair)	Based on weight and total IgE, SC every 2-4 wk	≥6 yr old; positive allergy testing (allergic asthma); IgE, 30–1500 IU/mL	Anti-IgE; prevents IgE from binding to its receptor on mast cells and basophils

IV: Intravenous, SC: subcutaneous, IgE: immunoglobulin E, wk: week, yr: year, IL: interleukin, ACE: absolute eosinophil count, FeNO: fractional nitric oxide

Patients should be prescribed carefully: are they asthmatics who could be controlled with low-dose ICS if used effectively, in which case the TH2 endotype is likely to be critical, or are they true severe therapy-resistant asthma (STRA), in which case numerous endotypes are likely to be important? The first priority is to establish who should be given Omalizumab and who should be given Mepolizumab, as these are the 2 biologicals approved for use in children.^65,66^

### Omalizumab

It is used to treat severe allergic asthma that does not respond to high doses of corticosteroids, and it’s also used to treat persistent spontaneous urticaria in some cases.^65^ Omalizumab was approved for use in pediatrics with severe asthma, which acts by interaction with the peripheral blood IgE and preventing their binding with the IgE receptor (FCεR1) on the surface of the basophil and mast cells, leading to pro-inflammatory mediators inhibition.^66^ Furthermore, Omalizumab indirectly inhibits the upregulation of FCR1.^67^ It does not bind to IgE that has already been bound by the FCεR1 on the surface of mast cells, basophils, and antigen-presenting dendritic cells, unlike typical anti-IgE antibodies.^68^

For children with allergic asthma and increased serum IgE, omalizumab can be prescribed as an additional therapy.^69^ It has been reported that Omalizumab has favorable outcomes in asthmatic children with high peripheral eosinophil counts, elevated serum periostin, FeNO >20 ppb, and multiple-allergic comorbidities.^70^ However, there are no sufficient data on the prediction of Omalizumab therapy response by validated biomarkers in children; therefore, further investigations are necessary.^71,72^

The effectiveness and safety of Omalizumab in pediatrics have been demonstrated by many randomized controlled trials.^73,74^ Pediatric trials have notably shown that the frequency of asthma attacks, hospitalization, and the necessity for oral corticosteroids (OCS) has been lowered with Omalizumab.^75,76^ In addition, Omalizumab greatly enhanced patients’ asthma management and quality of life (QOL).^77^ Finally, the number of seasonal exacerbations in patients who received Omalizumab was lower than that of the control.^75^ Many studies have shown that Omalizumab in children and adolescents is generally well-tolerated.^78-80^ In 10 studies with 3261 patients, Omalizumab was associated with a significant reduction in asthma attacks (OR [odds ratio]=0.55, 95% confidence interval [CI]: 0.42-0.60), with an absolute reduction rate of 16% to 26%. Moreover, hospital admission was observed to be reduced in 4 studies with 1824 (OR=0.16, 95% CI: 0.06-0.42), with an absolute reduction rate of 0.5% to 3%.^79^

Serious or life-threatening conditions related to the medication, such as anaphylaxis, have been observed in 0.2% of adolescents who received Omalizumab; however, there is no evidence of being exciting in children.^78,81^ The most common side events described in the literature are skin reactions and pain at the site of injection, which usually resolve quickly.^69^ In addition, there is no evidence that Omalizumab is associated with an elevated risk of cancer. Nevertheless, longitudinal studies on children are still needed to endorse favorable safety records.

### Mepolizumab

Mepolizumab was licensed as a supplemental maintenance medication for many conditions, including serious eosinophilic asthma, an eosinophilic phenotype, and asthma exacerbation history.^82^ Adults and children over 12 should take 100 mg, while children aged 6-11 should take 40 mg. Despite the lack of standardized response criteria, clinical and laboratory indicators have been proposed as prediction tools.^82^ The reduction in FEV1 value and the blood eosinophil count of 300 cells/L are now considered to be measures of responsiveness to Mepolizumab therapy. Furthermore, clinical predictors of response to therapy include improvements in QoL, exacerbations, and physical fitness.^83^

Mepolizumab was studied in patients with eosinophilic asthma who did not respond to medication in 2 trials that demonstrated a significant reduction in asthma exacerbations.^84^ Mepolizumab demonstrated a considerable decline in OCS use and a notable enhancement in the symptoms and lung function of patients.^85,86^ Further studies are needed to assess the role of Mepolizumab in children less than 12 years. Mepolizumab had a good safety profile and was shown to be well-tolerated in placebo-controlled trials.^87,88^ Respiratory infections, reactions at the injection site, fatigue, headaches, and asthma exacerbation were the most frequently described side events.^89^

### Dupilumab

It targets the IL-4 receptors that is released by CD4+ Th2 cells and enhance the generation of IgE and the recruitment of inflammatory cells.^90,91^ Moreover, the levels of T2 inflammation markers such as FeNO, eotaxin-3, and IgE demonstrated a significant reduction.^85,86,92^ In cases of moderate-to-severe asthma, it also enhances lung function. Eosinophils and FeNO in peripheral blood are efficient indicators of therapy response.^93^

Dupilumab is now licensed for adolescents and adults who have moderate to severe asthma with oral corticosteroid-dependent asthma or an eosinophilic phenotype.^94,95^ Dupilumab has a good safety record, with injection site reactions and transient blood eosinophilia being the most prevalent side effects, and it is now being assessed by Food and Drug Administration.^92^

### Selection of biologics for severe asthma

The best biological drug cannot be detected because there are no direct comparisons between them. In selecting certain biologics, it is essential to measure the mechanism of action of medication, comorbidities and drug cost, atopic state, serum levels of IgE and FeNO, and blood levels of eosinophil.^93^ Omalizumab may be first prescribed for allergic asthma patients. Anti-IL-5 therapy may be considered as a first-line treatment for eosinophilic asthma patients with a history of exacerbations.^94^ Dupilumab may be first used in severe asthmatic individuals with atopic dermatitis.^95^

It was suggested to use some factors, including inflammatory biomarkers, exacerbations, symptom onset, and associated-allergic tendencies, to establish a strategy for finding appropriate biologics.^96^ Furthermore, the algorithm must be updated regularly, taking into account recent research findings on outcome predictors and drug development. Additionally, using adult data and applying it to pediatric populations with asthma should be avoided, and more pediatric clinical studies are needed to accurately define the usage of biological therapy in severely asthmatic children.^63^

## 8. Other medications used for severe asthma

### Systemic corticosteroids

Several studies have shown that short-term OCS therapy (3 or 5 days) can reduce the intensity and duration of an asthma exacerbation in children.^97^ Oral corticosteroids therapy can be given to some children and adolescents for longer than a month daily or alternate daily. Despite being recommended in asthma guidelines, “maintenance” OCS has little evidence of effectiveness.^98^ Using OCS for short periods is known to cause side effects in children (sleep disturbance, vomiting, and behavior change)^99^ and for intervals longer than 14 days (susceptibility to infection, cushingoid features, growth retardation, and weight gain).^99,100^

### Intramuscular triamcinolone

Intramuscular triamcinolone therapy may help identify steroid-responsive asthma and treat severe asthma.^101^ The evidence is limited to case series using various dosages of triamcinolone.^102-104^ A study showed that triamcinolone therapy reduced blood eosinophil count and FeNO. The relative failure of triamcinolone in non-severe asthmatic children is likely owing to adequate baseline FEV1, mild symptoms, and limited sample size.^54^ Another study evaluated symptoms and physiological responses one month following triamcinolone administration. The Asthma Control Test showed better symptom scores and spirometry in children who received triamcinolone. Treatment decreased sputum eosinophilia, FeNO, and intensive care unit hospitalizations, but only in white children. Triamcinolone, like other asthma medications, has a variable response.^103^ For children and adolescents, it is appropriate to start a brief trial of triamcinolone therapy to see if symptoms respond to steroid treatment. If after 2 months of treatment there is no improvement or adverse effects arise, treatment may be terminated.

## 9. Impact of severe asthma on children’s quality of life

Severe asthma control and quality of life were also shown to be linked, according to the many studies. Research carried out in the United States found that patients with severe asthma who had insufficient management of their condition had clinically significant levels of behavioral problems.^105^ Another study found that the prevalence of emotional and behavioral problems among asthmatic adolescents was 20.6%, compared to 9% for nonasthmatic adolescents.^106^ In addition, anxiety, depression, and behavioral changes are more prevalent in uncontrolled asthma.^107,108^ Banjari et al,^109^ showed that among 106 Saudi children with severe asthma, 84% had poor asthma control. Children with uncontrolled asthma had a significantly worse quality of life (*p*<0.001). The psychological well-being of children with and without asthma control was comparable (*p*=0.58); however, both groups were negatively impacted. Therefore, they concluded that psychosocial well-being should be measured during clinic visits, in order to take a more holistic approach and enhance outcomes.

## 10. Requirements

### Severe pediatric asthma service goals

Proper assessment, enhancing self-management, controlling the triggers, reducing the comorbidities, and providing opportunities for high-quality research and training are essential.^110^ The assessment of severe asthma might be complicated by misdiagnosis and symptom misattribution. Therefore, objective confirmation of an asthma diagnosis by showing the defining characteristic of asthma is required.^111^ Many tests can be used to achieve this, including airway hyperresponsiveness, assessments of bronchodilator responsiveness, and variability of airflow over time.^112-114^ The airway hyperresponsiveness can be measured using hypertonic saline, mannitol, or methacholine, while bronchodilator responsiveness can be assessed using pre-and post-bronchodilator spirometry.^104,105^ In terms of airflow variability, peak expiratory flow readings or serial spirometry can be used. After confirming an asthma diagnosis, it is crucial to look for potential aggravating or coexisting variables that could make asthma management more difficult.^115^

Another important goal is to enhance self-management skills, which can directly improve asthma control.^116-117^ Self-monitoring, inhaler technique, written action plan, and medication adherence are critical skills for asthma management that should be targeted in a severe asthma service.^107,108^ Studies showed that early optimization of these skills is essential to achieve adequate control. In addition, it is critical to identify and assess potential trigger factors.^118^ Allergens, industrial pollutants, cigarettes, and recurrent infections are all triggers. Asthma control can be improved by removing these triggers.^59^ A severe asthma clinic’s structured multidisciplinary approach provides high-quality training for various healthcare providers.^110^

### The role of the multidisciplinary team

The minimum required team to run the severe asthma service includes a pediatric pulmonologist, pediatric nurse, and respiratory therapist. Further team members necessary for multidisciplinary care include speech pathologist, dietitian, physiotherapist, psychologist, gastroenterologist, pharmacist, and administrative support.^119^ Many specialties are required to confirm the diagnosis, including respiratory physician, pulmonary function scientist, and radiographer. Optimize self-management needs respiratory physicians and nurse specialists.^110^ Regarding the treatment of asthma triggers and comorbidities, pharmacists, respiratory physicians, advanced trainees, nurse specialists, dietitians, psychologists, gastroenterologists, sleep physicians, and physiotherapy are required.^120^ Each clinic should conduct a multidisciplinary case review meeting to evaluate patient progress. These meetings will improve the team-based approach and increase the skills of the clinicians, which in turn will enhance the patients’ outcomes.

### Facilities

A proper location to give drugs such as Omalizumab is also required.^121^ Adrenaline and other vital life-saving supplies need to be readily available at this place in the event of a medical emergency. Pharmacies should be close to doctors or an emergency response team. Telephone support should be available at all times in order to provide timely management of acute exacerbations or treatment-related adverse effects.^122^ Senior nursing professionals, advanced trainees, or registrars can provide this support in consultation with the respiratory physician. The use of a conference room with access to healthcare information for a multidisciplinary case review is highly suggested.^110^ To confirm the diagnosis, clinics, pulmonary function laboratories, and medical imaging are needed. Regarding the treatment of asthma triggers, a sleep laboratory, rapid access clinic, drug administration clinic, facilities for aspirin-sensitive asthma desensitization, sputum clearance devices, and thoracic radiology are required.^123^

## 10. Conclusion

The severity assessment of asthmatic children is dictated by the treatment step needed to control the patient’s symptoms. The evaluation approach to severe asthma can be simplified into 3 steps: i) Confirm diagnosis of asthma, using full clinical evaluation, pulmonary function tests, psychosocial assessment, and other investigations; ii) Check barriers for good control as poor adherence, poor techniques skills, and improper environment; and iii) Exclude comorbidities that significantly associated with exacerbation frequency. The best biological drug cannot be detected because there are no direct comparisons between them, as well as there are no efficient biomarkers for predicting or monitoring the treatment response. Regarding the service requirements, multifactorial services, including proper assessment, enhancing self-management, controlling the triggers, reducing the comorbidities, and providing opportunities for high-quality research and training are essential. The minimum required multidisciplinary team to run the severe asthma service includes a pediatric pulmonologist, pediatric nurse, and respiratory therapist. Further team members necessary for multidisciplinary care include speech pathologist, dietitian, physiotherapist, psychologist, gastroenterologist, pharmacist, and administrative support.

Finally, further epidemiological studies are required to assess the prevalence of severe asthma in Saudi children and identify the regular clinical practice used in primary healthcare centers in Saudi Arabia.
